# Surgery offers survival advantage over radiotherapy in patients who are 80 years and older with Stage I and II NSCLC: A retrospective cohort study of 7,045 patients

**DOI:** 10.3389/fsurg.2022.1018320

**Published:** 2022-10-04

**Authors:** Qiang Guo, Sheng Hu, Jiayue Ye, Lang Su, Silin Wang, Deyuan Zhang, Yang Zhang, Shengyu Qiu, Lingxiao Zhu, Liancheng Ruan, Bingen Wan, Sheng Zou, Wenxiong Zhang, Dongliang Yu, Jianjun Xu, Huiliang Zhang, Yiping Wei

**Affiliations:** ^1^ Department of Thoracic Surgery, The Second Affiliated Hospital of Nanchang University, Nanchang, China; ^2^ Department of Thoracic Surgery, XinSteel Center Hosptial, Xinyu, China

**Keywords:** treatment, surgery, radiation, survival, NSCLC, SEER

## Abstract

**Objective:**

Elderly people are less likely than younger patients to undergo curative surgery for early-stage lung cancer because of the greater risk of surgery and postoperative complications. We investigated the relationship between treatment modality and the risk of all-cause and lung cancer-specific mortality to compare the efficacy of surgical treatment with radiotherapy in patients with stage I and II non-small cell lung cancer (NSCLC) who were ≥80 years old.

**Methods:**

We extracted data from the most recent Surveillance, Epidemiology, and End Results 9 registry study database (2010–2017). We mainly selected patients with stage I and II NSCLC who were ≥80 years old, and after screening, 7,045 cases were selected for our study. We used univariate analysis, stratified analysis, and multiple regression equation analysis to examine all-cause mortality and lung cancer-specific mortality in different treatment modalities. The overall and stratified populations' survival curves were plotted using the Kaplan–Meier method. The competing risk regression method of Fine and Gray was used to estimate mortality specific to lung cancer.

**Results:**

In the fully adjusted model, all-cause mortality was 1.97 times higher in the radiotherapy-only group (hazard ration (HR) = 1.97, 95% confidence interval (CI) = 1.81–2.14, *p* < 0.0001) than in the surgery-only group. The lung cancer-specific mortality rate was 1.22 times higher in the radiotherapy-only group (HR = 1.22, 95% CI = 1.13–1.32, *p* < 0.0001) than in the surgery-only group. The median overall survival (OS) in the surgery-only, radiation therapy-only, surgery plus radiation therapy, and no-treatment groups were 58 months, 31 months, 36 months, and 10 months, respectively. Median lung cancer-specific survival was 61 months, 32 months, 38 months, and 11 months, respectively. The surgery-only group had the highest 1-year OS (0.8679,95% CI = 0.8537–0.8824) and 5-year OS (0.4873, 95% CI = 0.4632–0.5126).

**Conclusions:**

Surgery had a higher overall and lung cancer-specific survival rate than radiotherapy and no treatment in the elderly early-stage NSCLC population. For patients with stage I and stage II NSCLC at advanced ages, surgical treatment might have a greater potential survival benefit.

## Introduction

In terms of incidence and mortality, lung cancer is the most prevalent cancer in the world (1). With increasing average age, advanced aged lung cancer is becoming more common. The median age of onset of lung cancer is 70 years old, making it the highest cause of cancer-related death among older patients (2, 3). Therapy is dependent on the findings of trials performed on younger patients, and older patients are underrepresented in clinical trials, despite having a higher death rate (2, 4). The physiological changes caused by aging organ function and organ reserve might lead to decreased tolerance of treatment and increased toxicity, in addition to more comorbidities, in elderly patients, which are associated with multiple drug interactions, all of which increase the risk of mortality and postoperative complications of surgical treatment (5). Many medical professionals avoid surgery or limit it depending on age because of the higher risk of postoperative complications and surgery in senior patients (6). Consequently, elderly people with early-stage lung cancer are less likely than younger patients to receive curative surgery (7–9). However, with the increasing economic level and developments in medical treatment, the physical condition of patients aged 80 years and above has also improved compared with previous generations, thus there might be a problem of under-representation in using the results from previous studies as clinical guidance.

In addition, one study found that radiotherapy was associated with a higher overall survival (OS) and a lower rate of treatment-related adverse events than surgical treatment for elderly patients with operable stage I non-small cell lung cancer (NSCLC) (10). However, several studies have also shown that older patients who underwent surgery for early-stage lung cancer with acceptable toxicity had similar cancer-related mortality and OS to younger patients (11, 12). These results were from an earlier, small single-center report and might not reflect the most recent high-volume multicenter experience.

The Surveillance, Epidemiology, and End Results (SEER) database of the National Cancer Institute collects cancer diagnosis, treatment, and survival data for approximately 30 percent of the U.S. population. SEER is an important population-based resource for examining the diagnostic implications of pathology across demographic characteristics, geographical regions, and time, and has become a unique research resource for oncology practices in the United States. It contains incidence, survival, and death data for many histopathological cancer subtypes, and information on molecular typing is expanding. This study investigated the relationship between treatment modality and the risk of all cause and lung cancer-specific mortality in 7,405 patients with stage I and II NSCLC patients aged 80 years or older based on data from the SEER 9 database from 2010 to 2017 to compare the efficacy of surgical treatment with radiotherapy in these patients.

## Materials and methods

### Data source

We extracted data from the most recent SEER9 registry study database (submitted in November 2020). The database includes data from 1975 to 2018. Data from the SEER9 registries, including those in San Francisco-Oakland, Connecticut, Detroit (metropolitan), Hawaii, Iowa, New Mexico, Seattle (Puget Sound), Utah, and Atlanta (metropolitan), are included in the SEER9 database. In total, the SEER9 database includes data on 9.4% of the population of the United States (based on the 2010 Census). The Ethics Committee of the Second Affiliated Hospital of Nanchang University in China approved the study protocol. The patients could not be identified; therefore, the Ethics Committee of the Second Affiliated Hospital of Nanchang University decided not to review this study.

### Cohort selection

**Figure 1:**
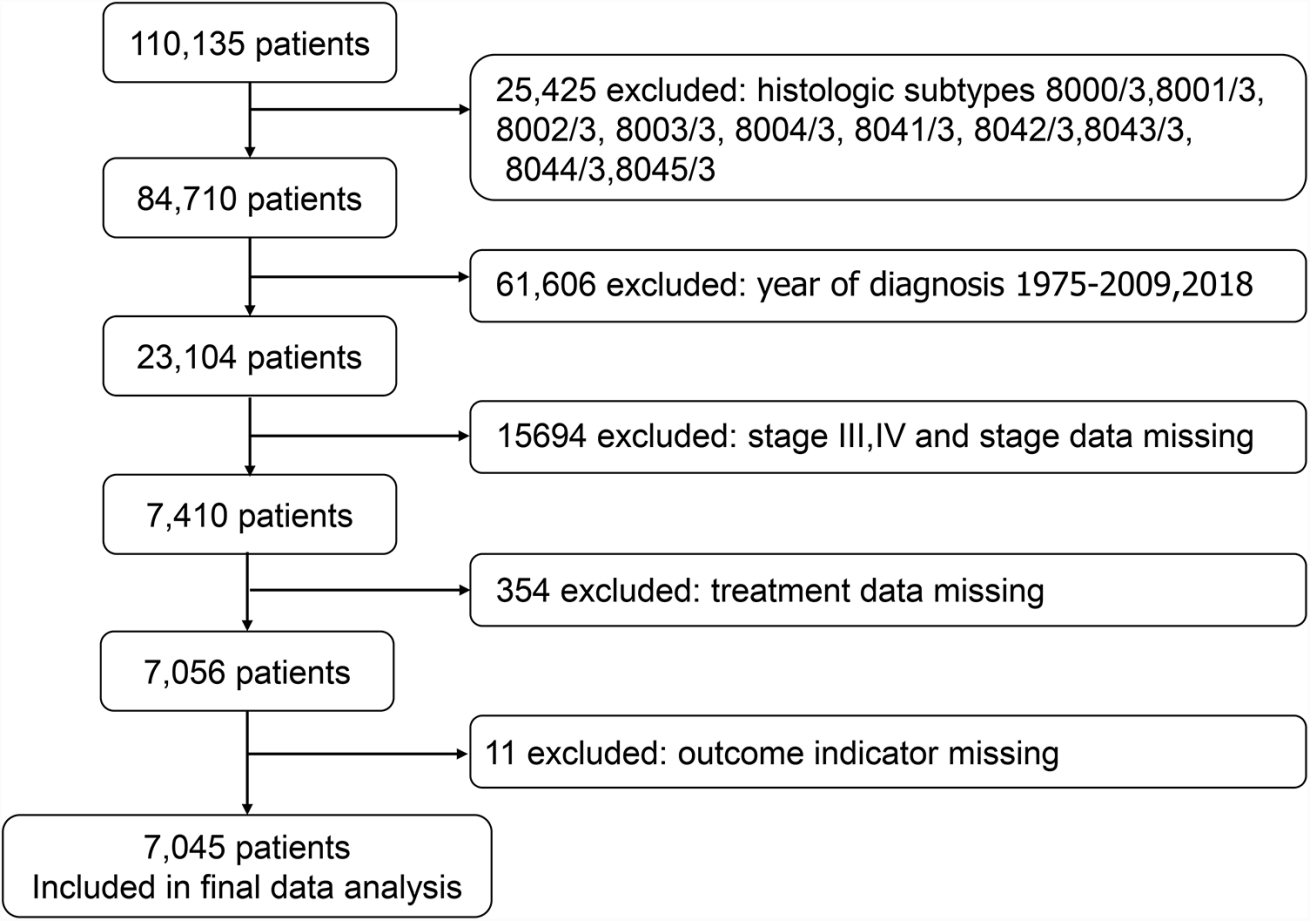
Flowchart used to screen the participants.

SEER*Stat version 8.3.9.2 (seer.cancer.gov/seerstat) was used to generate the case list. We extracted cases of lung cancer in patients aged 80 years or older. The case list contained information on the following variables: age, ethnicity, sex, year of diagnosis, primary site, grade, laterality, histology, stage group, T stage, N stage, treatment, sequence number, number of tumors, marital status, and chemotherapy. Ethnicity was recorded as white, black, and other races. Data on treatment were also extracted, including radiation (yes, no) and surgery (yes, no) and chemotherapy (yes, no). We identified 110,135 cases of lung cancer in patients aged 80 years or older. We excluded 25,425 cases with the following histological subtypes: 8000/3, 8001/3, 8002/3, 8003/3, 8004/3, 8041/3, 8042/3, 8043/3, 8044/3, and 8045/3, because these types were unknown histological types and small cell lung cancer. We excluded 61,606 cases with a diagnosis year of 1975–2009 as well as cases from 2018, and only cases with diagnosis years 2010–2017 were retained. The Union for International Cancer Control (UICC) 7th edition lung cancer staging was used for tumor staging. We excluded 15,694 patients with stages III and IV and patients with missing staging data, as well as 365 cases with missing outcome index data and treatment modality data. Ultimately, 7,045 cases were selected for the study (Figure 1).

### Vital status

The status of the patients at the most recent follow-up was extracted using SEER9's “cause of death (COD) to site recode” variable. Based on this data, we divided all patients into three groups: (1) Those who survived; (2) those who died from lung cancer; and (3) those who died from other reasons. The primary outcome was total mortality. The secondary outcomes were lung cancer specific mortality and non-lung cancer specific mortality. From the date of diagnosis to the date of the last follow-up appointment, temporal information was extracted using the variable “survival months.” By deducting the date of diagnosis from the date of last contact, the SEER*stat program calculates survival time (in months) (study cut-off date). Days in a month = 365.24/12. The study cut-off date was 31 December 2018.

### Statistical analysis

Patients were categorized based on the types of therapies they received, including no treatment, surgery-only, radiotherapy-only, and surgery + radiotherapy. No treatment was defined as no surgery or radiotherapy, with or without chemotherapy. We defined OS as the period of time between the diagnosis of lung cancer and the date of death from any cause, and we defined specific survival as the interval between the diagnosis of lung cancer and the date of death from lung cancer. Univariate analysis (unadjusted) was used to identify covariates affecting mortality, and stratified analysis (adjusted) was used to find the effect of each type of population on mortality. The Kaplan–Meier method (KM) was used to plot survival curves for patients older than 80 years with early-stage NSCLC according to the four treatment modalities. KM all-cause survival curves and lung cancer-specific survival curves stratified by variables such as sex were used to assess the effect of treatment modality on patient survival in different populations. Cox proportional risk analysis was used to examine age, ethnicity, sex, year of diagnosis, primary site, grade, laterality, histology, stage group, T stage, N stage, treatment, sequence number, number of tumors, marital status, and chemotherapy on all-cause mortality and lung cancer-specific mortality in advanced age patients with early-stage NSCLC. The competing risk regression method of Fine and Gray was used to estimate mortality specific to lung cancer (13). Empower (R) (www.empowerstats.com, X/Y solutions, Inc. Boston, MA, USA) and R version 3.6.3 (http://www.R-project.org) were used for all analyses. Empower Stats is a statistical software based on the R language for data analysis. The software has powerful data processing functions, as well as comprehensive analysis functions. The agreed cut off for statistical significance was *p* < 0.05.

## Results

### Baseline characteristics of the study participants by treatment modality

About 30% of the participants received surgery only, about 46% received radiotherapy only, about 1% received surgery plus radiotherapy, and about 23% of the patients received neither surgery nor radiotherapy (Table 1). Participants who underwent surgery were more likely to be younger, have adenocarcinoma, and be married. There was a strong correlation between treatment modality and tumor grade: 20.24% of those treated with radiotherapy only were grade II, while the percentage of grade II was 44.67% and 39.51% among those treated with surgery only and those treated with surgery plus radiotherapy, respectively. Those treated with surgery only had an earlier year of diagnosis compared with those treated with radiotherapy only.

**Table 1:** Baseline characteristics of the study participants by treatment method.

Treatment	Surgery only	Radiation only	Surgery + Radiation	No treatment	*p*-value
Age					<0.001
80–84 years	1,689 (78.59%)	1,838 (57.31%)	63 (77.78%)	770 (47.89%)	
85–89 years	424 (19.73%)	1,111 (34.64%)	17 (20.99%)	599 (37.25%)	
90–94 years	34 (1.58%)	238 (7.42%)	1 (1.23%)	191 (11.88%)	
95–100 years	2 (0.09%)	20 (0.62%)	0 (0.00%)	48 (2.99%)	
Ethnicity					<0.001
White	1,838 (85.53%)	2,759 (86.03%)	71 (87.65%)	1,313 (81.65%)	
Black	113 (5.26%)	197 (6.14%)	2 (2.47%)	122 (7.59%)	
Other	198 (9.21%)	251 (7.83%)	8 (9.88%)	173 (10.76%)	
Sex					0.017
Male	1,042 (48.49%)	1,543 (48.11%)	40 (49.38%)	704 (43.78%)	
Female	1,107 (51.51%)	1,664 (51.89%)	41 (50.62%)	904 (56.22%)	
Year of diagnosis					<0.001
2010–2013	1,200 (55.84%)	1,365 (42.56%)	47 (58.02%)	856 (53.23%)	
2014–2017	949 (44.16%)	1,842 (57.44%)	34 (41.98%)	752 (46.77%)	
Primary site					<0.001
Upper lobe	1,202 (55.93%)	1,849 (57.66%)	39 (48.15%)	908 (56.47%)	
Middle lobe	149 (6.93%)	143 (4.46%)	5 (6.17%)	77 (4.79%)	
Lower lobe	771 (35.88%)	1,124 (35.05%)	36 (44.44%)	527 (32.77%)	
Main bronchus	3 (0.14%)	26 (0.81%)	1 (1.23%)	23 (1.43%)	
Unknow	24 (1.12%)	65 (2.03%)	0 (0.00%)	73 (4.54%)	
Grade					<0.001
I	430 (20.01%)	291 (9.07%)	8 (9.88%)	146 (9.08%)	
II	960 (44.67%)	649 (20.24%)	32 (39.51%)	206 (12.81%)	
III	538 (25.03%)	630 (19.64%)	33 (40.74%)	238 (14.80%)	
IV	20 (0.93%)	14 (0.44%)	0 (0.00%)	11 (0.68%)	
Unknown	201 (9.35%)	1,623 (50.61%)	8 (9.88%)	1,007 (62.62%)	
Laterality					0.040
Left- origin of primary	894 (41.60%)	1,436 (44.78%)	33 (40.74%)	712 (44.28%)	
Right- origin of primary	1,255 (58.40%)	1,769 (55.16%)	48 (59.26%)	892 (55.47%)	
Unknown	0 (0.00%)	2 (0.06%)	0 (0.00%)	4 (0.25%)	
Histology					<0.001
Squamous cell neoplasms	537 (24.99%)	1,088 (33.93%)	24 (29.63%)	418 (26.00%)	
Adenomas and adenocarcinomas	1,221 (56.82%)	1,529 (47.68%)	48 (59.26%)	693 (43.10%)	
Other	391 (18.19%)	590 (18.40%)	9 (11.11%)	497 (30.91%)	
Stage					<0.001
I	1,690 (78.64%)	2,514 (78.39%)	35 (43.21%)	1,032 (64.18%)	
II	459 (21.36%)	693 (21.61%)	46 (56.79%)	576 (35.82%)	
T stage					<0.001
T1	1,024 (47.65%)	1,801 (56.16%)	19 (23.46%)	692 (43.03%)	
T2	940 (43.74%)	1,056 (32.93%)	37 (45.68%)	624 (38.81%)	
T3	185 (8.61%)	350 (10.91%)	25 (30.86%)	292 (18.16%)	
N stage					<0.001
N0	1,985 (92.37%)	3,029 (94.45%)	65 (80.25%)	1,453 (90.36%)	
N1	164 (7.63%)	178 (5.55%)	16 (19.75%)	155 (9.64%)	
Sequence number					0.008
First/only primary	1,216 (56.58%)	1,768 (55.13%)	49 (60.49%)	968 (60.20%)	
Second/higher-order primary	933 (43.42%)	1,439 (44.87%)	32 (39.51%)	640 (39.80%)	
Number of tumors					<0.001
1	1,021 (47.51%)	1,568 (48.89%)	42 (51.85%)	895 (55.66%)	
2	739 (34.39%)	1,055 (32.90%)	27 (33.33%)	488 (30.35%)	
3+	389 (18.10%)	584 (18.21%)	12 (14.81%)	225 (13.99%)	
Marital status					<0.001
Married	1,072 (49.88%)	1,331 (41.50%)	45 (55.56%)	614 (38.18%)	
Widowed	733 (34.11%)	1,287 (40.13%)	27 (33.33%)	688 (42.79%)	
Other	344 (16.01%)	589 (18.37%)	9 (11.11%)	306 (19.03%)	
Chemotherapy					<0.001
No	2,061 (95.91%)	2,879 (89.77%)	57 (70.37%)	1,489 (92.60%)	
Yes	88 (4.09%)	328 (10.23%)	24 (29.63%)	119 (7.40%)	

Continuous variables are presented as the mean ± SD; Categorical variables are presented as *n* (%).

### Univariate analysis of the association between treatment modality and mortality

In the unadjusted univariate analysis (Table 2), the radiation-only group had higher all-cause mortality (hazard ratio (HR) = 1.93, 95% confidence interval (CI) = 1.79–2.08, *p* < 0.0001) and lung cancer-specific mortality (HR = 1.49, 95% CI = 1.40–1.60, *p* < 0.0001), and the no-treatment group had the highest all-cause mortality (HR = 4.23, 95% CI = 3.90–4.59, *p* < 0.0001) and lung cancer-specific mortality (HR = 1.90, 95% CI = 1.73–2.08, *p* < 0.0001), as referenced by the surgery-only population. All-cause mortality in the surgery plus radiotherapy group (HR = 1.61, 95% CI = 1.23–2.12, *p* = 0.0006) was between the surgery-only and radiotherapy-only groups, while their lung cancer-specific mortality (HR = 0.98, 95% CI = 0.72–1.32, *p* = 0.8811) was similar to that in the surgery-only population, but was not statistically significant. All-cause mortality was also related to age, sex, year of diagnosis, primary site, grade, histology, stage group, T stage, N stage, number of tumors, marital status, and chemotherapy. Lung cancer-specific mortality was also associated with age, sex, year of diagnosis, histology, N stage, and chemotherapy.

**Table 2:** Crude univariate analysis of the association between treatment method and mortality.

Exposure	Statistics	Hazard Ratio (95% CI) *p*-value	
		All-cause mortality	Lung cancer-specific mortality
Age			
80–84 years	4,360 (61.89%)	1	1
85–89 years	2,151 (30.53%)	1.31 (1.23, 1.40) <0.0001	1.18 (1.10, 1.26) <0.0001
90–94 years	464 (6.59%)	1.82 (1.63, 2.03) <0.0001	1.45 (1.27, 1.65) <0.0001
95–100 years	70 (0.99%)	2.76 (2.14, 3.57) <0.0001	1.45 (0.95, 2.21) 0.0821
Ethnicity			
White	5,981 (84.90%)	1	1
Black	434 (6.16%)	1.05 (0.93, 1.19) 0.4056	1.04 (0.91, 1.18) 0.5835
Other	630 (8.94%)	0.85 (0.76, 0.94) 0.0023	0.94 (0.85, 1.04) 0.2449
Sex			
Male	3,329 (47.25%)	1	1
Female	3,716 (52.75%)	0.76 (0.72, 0.81) <0.0001	0.88 (0.82, 0.93) <0.0001
Year of diagnosis			
2010–2013	3,468 (49.23%)	1	1
2014–2017	3,577 (50.77%)	0.92 (0.87, 0.98) 0.0108	4.52 (4.17, 4.89) <0.0001
Primary site			
Upper lobe	3,998 (56.75%)	1	1
Middle lobe	374 (5.31%)	0.81 (0.71, 0.94) 0.0036	0.92 (0.80, 1.05) 0.1920
Lower lobe	2,458 (34.89%)	1.04 (0.97, 1.10) 0.2522	1.03 (0.97, 1.10) 0.3127
Main bronchus	53 (0.75%)	2.15 (1.61, 2.88) <0.0001	0.85 (0.51, 1.41) 0.5320
Unknown	162 (2.30%)	1.88 (1.57, 2.24) <0.0001	1.12 (0.88, 1.44) 0.3592
Grade			
I	875 (12.42%)	1	1
II	1,847 (26.22%)	1.50 (1.34, 1.68) <0.0001	1.05 (0.96, 1.16) 0.2972
III	1,439 (20.43%)	2.00 (1.79, 2.24) <0.0001	1.09 (0.98, 1.22) 0.0938
IV	45 (0.64%)	2.31 (1.63, 3.25) <0.0001	1.01 (0.65, 1.58) 0.9546
Unknown	2,839 (40.30%)	2.21 (1.99, 2.45) <0.0001	1.53 (1.40, 1.68) <0.0001
Laterality			
Left-origin of primary	3,075 (43.65%)	1	1
Right-origin of primary	3,964 (56.27%)	0.97 (0.92, 1.03) 0.2977	1.01 (0.95, 1.07) 0.8166
Unknown	6 (0.09%)	3.13 (1.30, 7.53) 0.0109	2.77 (0.69, 11.10) 0.1498
Histology			
Squamous cell neoplasms	2,067 (29.34%)	1	1
Adenomas and adenocarcinomas	3,491 (49.55%)	0.60 (0.57, 0.65) <0.0001	0.83 (0.77, 0.89) <0.0001
Other	1,487 (21.11%)	0.92 (0.85, 0.99) 0.0363	1.12 (1.02, 1.22) 0.0125
Stage			
I	5,271 (74.82%)	1	1
II	1,774 (25.18%)	1.76 (1.66, 1.88) <0.0001	0.93 (0.86, 1.01) 0.0952
T stage			
T1	3,536 (50.19%)	1	1
T2	2,657 (37.71%)	1.45 (1.36, 1.54) <0.0001	0.95 (0.89, 1.02) 0.1349
T3	852 (12.09%)	2.03 (1.86, 2.22) <0.0001	0.96 (0.86, 1.07) 0.4869
N stage			
N0	6,532 (92.72%)	1	1
N1	513 (7.28%)	1.43 (1.29, 1.58) <0.0001	0.86 (0.75, 0.99) 0.0321
Treatment			
Surgery only	2,149 (30.50%)	1	1
Radiation only	3,207 (45.52%)	1.93 (1.79, 2.08) <0.0001	1.49 (1.40, 1.60) <0.0001
Surgery + Radiation	81 (1.15%)	1.61 (1.23, 2.12) 0.0006	0.98 (0.72, 1.32) 0.8811
No treatment	1,608 (22.82%)	4.23 (3.90, 4.59) <0.0001	1.90 (1.73, 2.08) <0.0001
Sequence number			
First/only primary	4,001 (56.79%)	1	1
Second/higher-order primary	3,044 (43.21%)	1.04 (0.98, 1.10) 0.2139	1.08 (1.01, 1.15) 0.0146
Number of tumors			
1	3,526 (50.05%)	1	1
2	2,309 (32.78%)	0.97 (0.91, 1.03) 0.2977	0.97 (0.91, 1.04) 0.3566
3+	1,210 (17.18%)	0.91 (0.84, 0.99) 0.0260	1.02 (0.94, 1.11) 0.6431
Marital status			
Married	3,062 (43.46%)	1	1
Widowed	2,735 (38.82%)	1.08 (1.01, 1.15) 0.0205	1.01 (0.94, 1.08) 0.7915
Other	1,248 (17.71%)	1.04 (0.96, 1.13) 0.3266	1.07 (0.99, 1.17) 0.1011
Chemotherapy			
No	6,486 (92.07%)	1	1
Yes	559 (7.93%)	1.21 (1.10, 1.34) 0.0002	0.85 (0.75, 0.97) 0.0135

### Stratified analysis of the association between treatment modality and mortality

In the adjusted stratified analysis (Supplementary Table S1), in almost all strata of the population, the surgery-only group had the lowest mortality, the surgery plus radiotherapy group had higher mortality than the surgery-only group, the radiotherapy-only group had higher mortality than the surgery-plus-radiotherapy group, and the no-treatment group had the highest mortality. However, there were some differences in the results among those who received chemotherapy, in which all-cause mortality was slightly higher in the surgery plus radiotherapy group (HR = 2.19, 95% CI = 1.25–3.85, *p* = 0.0062) than in the radiotherapy-only group (HR = 2.02, 95% CI = 1.40–2.89, *p* = 0.0001). The specific adjusted variables are detailed in Supplementary Table S1.

### Multivariate analysis of the association between treatment modality and mortality

In the multiple regression analysis, all-cause mortality and lung cancer-specific mortality were significantly lower in the surgery-only group than in the other groups (Table 3). In the analysis with all-cause mortality as the outcome variable, both in the unadjusted model and in models I and II adjusted for sociodemographic and clinical variables, the groups with the lowest to highest mortality rates were, in descending order: the surgery-only group, the surgery plus radiotherapy group, the radiotherapy-only group, and the no-treatment group. In the fully adjusted model, all-cause mortality was 1.97 times higher in the radiotherapy-only group (HR = 1.97, 95% CI = 1.81–2.14, *p* < 0.0001) than in the surgery-only group and 4.37 times higher in the no-treatment group (HR = 4.37, 95% CI = 3.98–4.79, *p* < 0.0001) than in the surgery-only group. The surgery plus radiotherapy group (HR = 1.29, 95% CI = 0.98–1.70, *p* = 0.0666) was 1.29 times higher than the surgery-only group, but the result was statistically less significant.

**Table 3:** Multivariate analysis of the association between treatment method and mortality.

Exposure	Hazard Ratio (95% CI) *p*-value		
	Non-adjusted	Adjusted I	Adjusted II
All-cause mortality			
Treatment			
Surgery only	1	1	1
Radiation only	1.93 (1.79, 2.08) <0.0001	1.88 (1.74, 2.03) <0.0001	1.97 (1.81, 2.14) <0.0001
Surgery + Radiation	1.61 (1.23, 2.12) 0.0006	1.58 (1.20, 2.08) 0.0010	1.29 (0.98, 1.70) 0.0666
No treatment	4.23 (3.90, 4.59) <0.0001	4.19 (3.86, 4.56) <0.0001	4.37 (3.98, 4.79) <0.0001
Cause of death			
Lung cancer-specific mortality			
Surgery only	1	1	1
Radiation only	1.49 (1.40, 1.60) <0.0001	1.47 (1.37, 1.57) <0.0001	1.22 (1.13, 1.32) <0.0001
Surgery + Radiation	0.98 (0.72, 1.32) 0.8811	0.96 (0.71, 1.30) 0.7889	0.92 (0.68, 1.25) 0.5969
No treatment	1.90 (1.73, 2.08) <0.0001	1.89 (1.72, 2.07) <0.0001	1.77 (1.60, 1.97) <0.0001

Non-adjusted model adjusted for: None. Adjusted model I adjusted for: Age; Race; Sex. Adjusted model II adjusted for: Age; Race; Sex; Year of diagnosis; Primary site; Grade; Laterality; Histology; Stage; T stage; N stage; Sequence number; Number of tumors; Marital status; Chemotherapy.

In the analysis of lung cancer-specific mortality as an outcome variable, the groups with the lowest to highest mortality rates were, in descending order, the surgery-only group, the radiotherapy-only group, and the untreated group. The surgery plus radiotherapy group had no statistically significant HRs because of the disproportionately low number of events. In the fully adjusted model, the lung cancer-specific mortality rate was 1.22 times higher in the radiotherapy-only group (HR = 1.22, 95% CI = 1.13–1.32, *p* < 0.0001) than in the surgery-only group and 1.77 times higher in the no-treatment group (HR = 1.77, 95% CI = 1.60–1.97, *p* < 0.0001) than in the surgery-only group. The specific adjustment variables are detailed in Table 3.

### Overall survival and lung cancer-specific survival for people with different treatment modalities

In the survival analysis by the KM method, median survival was highest in the surgery-only group (Table 4). The median OS in the surgery-only, radiation therapy-only, surgery plus radiation therapy, and no-treatment groups was 58 (95% CI = 56–62) months, 31 (95% CI = 29–32) months, 36 (95% CI = 27–52) months, and 10 (95% CI = 9–11) months, respectively, and the median lung cancer-specific survival was 61 (95% CI = 57–67) months, 32 (95% CI = 30–34) months, 38 (95% CI = 25–56) months, and 11 (95% CI = 10–13) months, respectively.

**Table 4:** Overall survival and lung cancer-specific survival for people treated using different methods.

Treatment	Surgery only	Radiation only	Surgery + Radiation	No treatment
Over survival				
*N*	2,149	3,207	81	1,608
Median survival (95% CI; months)	58 (56–62)	31 (29–32)	36 (27–52)	10 (9–11)
1-year survival (95% CI)	86.79% (85.37%–88.24%)	78.04% (76.62%–79.48%)	80.25% (72.03%–89.40%)	44.94% (43.56%–47.44%)
5-year survival (95% CI)	48.73% (46.32%–51.26%)	22.84% (21.02%–24.82%)	31.92% (22.12%–46.07%)	9.46% (7.91%–11.32%)
Lung cancer-special survival				
*N*	2,149	3,207	81	1,608
Median survival (95% CI, months)	61 (57–67)	32 (30–34)	38 (25–56)	11 (10–13)
1-year survival (95% CI)	87.65% (86.09%–89.23%)	78.95% (77.11%–80.84%)	80.95% (71.82%–91.25%)	46.89% (43.48%–50.56%)
5-year survival (95% CI)	50.25% (47.54%–53.11%)	24.62% (22.20%–27.30%)	31.05% (20.09%–48.00%)	11.02% (8.67%–14.00%)

CI, Confidence interval.

**Figure 2:**
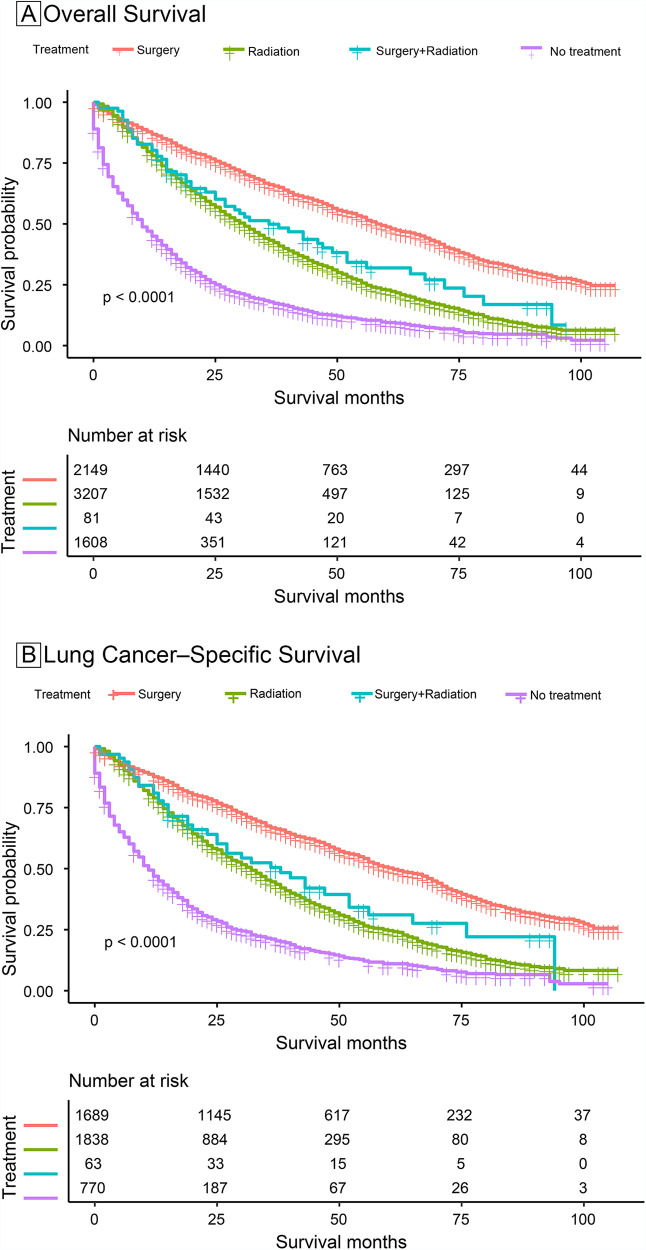
Survival stratified by treatment modalities among patients ≥80 years old with NSCLC. (**A**) Overall survival; (**B**) Lung cancer-specific survival.

The surgery-only group had the highest OS rates (Table 4 and Figure 2A). The 1-year OS rates in the surgery-only, radiotherapy-only, surgery plus radiotherapy, and no treatment groups were 0.8679 (95% CI = 0.8537–0.8824), 0.7804 (95% CI = 0.7662–0.7948), 0.8025 (95% CI = 0.7203–0.8940), and 0.4494 (95% CI = 0.4256–0.4744), respectively, and the 5-year OS rates were 0.4873 (95% CI = 0.4632–0.5126), 0.2284 (95% CI = 0.2102–0.2482), 0.3192 (95% CI = 0.2212–0.4607), and 0.0946 (95% CI = 0.0791–0.1132), respectively.

The surgery-only group had the highest rate for lung cancer-specific survival (Table 4 and Figure 2B). The lung cancer-specific survival rates at 1-year in the surgery-only, radiotherapy-only, surgery plus radiotherapy, and no treatment groups were 0.8765 (95% CI = 0.8609–0.8923), 0.7895 (95% CI = 0.7711–0.8084), 0.8095 (95% CI = 0.7181–0.9125), and 0.4689 (95% CI = 0.4348–0.5056), respectively, and the lung cancer-specific survival rates for lung cancer at 5 years were 0.5025 (95% CI = 0.4754–0.5311), 0.2462 (95% CI = 0.2220–0.2730), 0.3105 (95% CI = 0.2009–0.4800), and 0.1102 (95% CI = 0.0867–0.1400), respectively. To further analyze the survival of patients receiving different treatment modalities in different populations, we plotted stratified KM curves.

**Figure 3:**
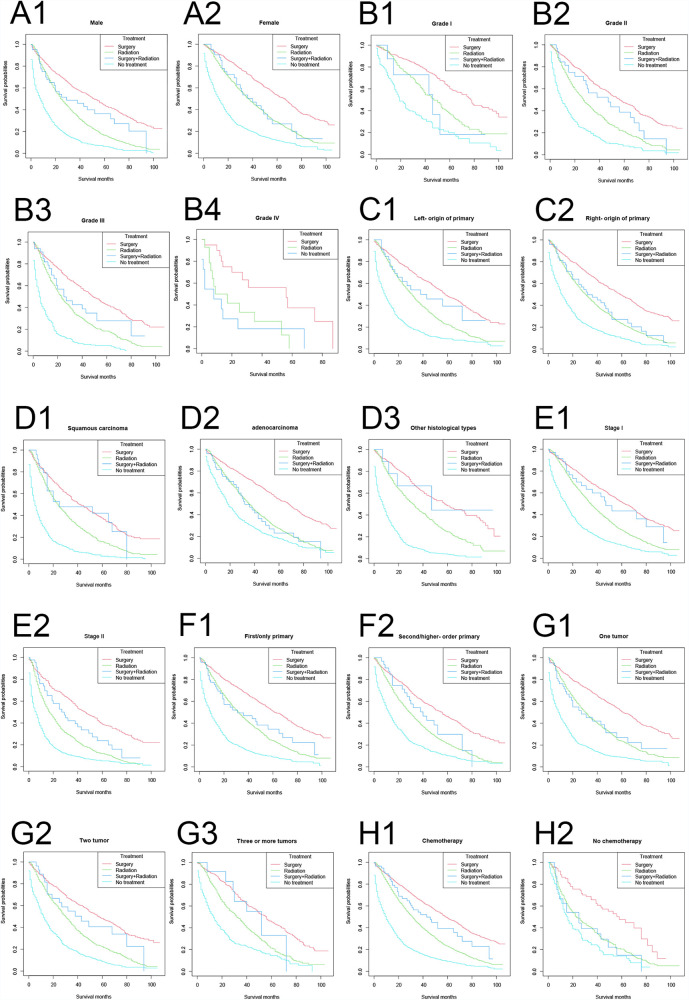
Overall survival stratified by treatment modalities among patients ≥80 years old with NSCLC in different stratifications. (A1–A2) Stratified by sex; (B1–B4) stratified by grade; (C1–C2) stratified by laterality; (D1–D3) stratified by histology; (E1–E2) stratified by stage; (F1–F2) stratified by sequence number; (G1–G3) stratified by number of tumors; (H1–H2) stratified by chemotherapy.

The longest OS in vast majority of the strata were, in descending order: the surgery-only group, the surgery plus radiotherapy-group, the radiotherapy only group, and the no treatment group (Figure 3). Among the female, adenocarcinoma, and no-chemotherapy populations, the OS curves in the surgery plus radiotherapy group overlapped with those in the radiotherapy group.

**Figure 4:**
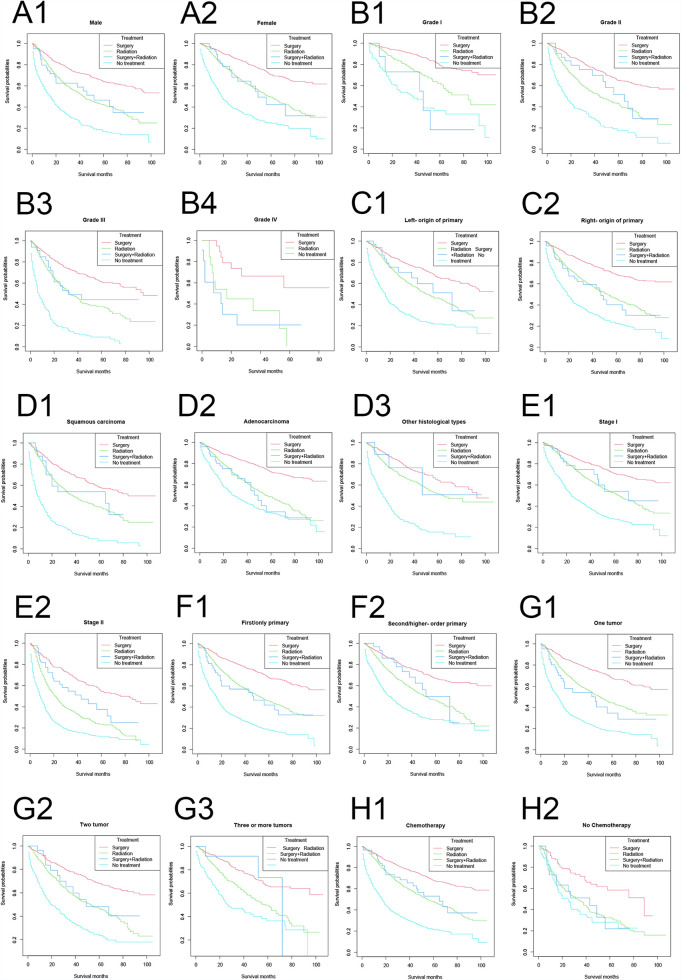
Lung cancer-specific survival stratified by treatment modalities among patients ≥80 years old with NSCLC in different stratifications. (A1–A2) Stratified by sex; (B1–B4) stratified by grade; (C1–C2) stratified by laterality; (D1–D3) stratified by histology; (E1–E2) stratified by stage; (F1–F2) stratified by sequence number; (G1–G3) stratified by number of tumors; (H1–H2) stratified by chemotherapy.

In vast majority of the strata, the lung cancer-specific survival rates were, in descending order: the surgery-only group, the surgery plus radiotherapy group, the radiotherapy-only group, and the no treatment group (Figure 4). Among the female, right primary, adenocarcinoma, and untreated populations, the lung cancer-specific survival curves in the surgery plus radiotherapy group overlapped with those in the radiotherapy-only group; and in the population with lung cancer as the first primary cancer and one tumor, the lung cancer-specific survival rates in the surgery plus radiotherapy group were lower than those in the radiotherapy group.

### Competing risk model analysis of the relationship between treatment modality and mortality

In the adjusted competing risk model, no significant difference was found in the non-lung cancer mortality risk by treatment modality, while the surgery-only group had the lowest risk of death because of lung cancer (Table 5). In the analysis of non-lung cancer death as an outcome indicator, the risk of death in the surgery-only group, the surgery plus radiation therapy group, and the untreated group was 1.1052 (95% CI = 0.9706–1.2586, *p* = 0.1312), 0.9796 (95% CI = 0.5893–1.6286, *p* = 0.9368), and 1.1693 (95% CI = 1.0001–1.3671, *p* = 0.0498), respectively. Using the surgery-only group as a reference, the HR values for all treatment modalities were not statistically significant, except for the untreated group, in which the HR values were slightly different from those of the surgery-only group.

**Table 5:** Treatment and cause-specific mortality in the advanced age early stage non-small cell lung cancer cohort.

Treatment	Death (not attributable to lung cancer)		Death (attributable to lung cancer)	
	Deaths (N)	HR (95% CI) *p*-value	Deaths (N)	HR (95% CI) *p*-value
Surgery only	513	1 (Ref)	557	1 (Ref)
Radiation only	881	1.11 (0.97–1.26) 0.13	1,249	4.11 (4.11, 4.11) <0.0001
Surgery + Radiation	17	0.98 (0.59–1.636) 0.94	37	2.71 (2.71–2.71) <0.0001
No treatment	467	1.17 (1.00–1.38) 0.05	922	25.05 (25.05–25.05) <0.0001

HŖ hazard ratio; CI, confidence interval. Adjusted by age, race, sex, primary site, grade, laterality, histology, stage group, number of tumors, marital status, and chemotherapy.

In the analysis using death from lung cancer as the outcome indicator, the risk of death from lung cancer in the surgery-only group, the surgery-plus-radiotherapy group, and the untreated group were 4.11 times (95% CI = 4.1064–4.1064, *p* < 0.0001), 2.71 times (95% CI = 2.7130–2.7130, *p* < 0.0001), and 25.05 times (95% CI = 25.0479–25.0479, *p* < 0.0001). The specific adjustment variables are detailed in Table 5.

## Discussion

In this retrospective cohort analysis based on the SEER 2010–2017 database, which included both stage I and stage II NSCLC populations aged 80 years and older, we found that after adjusting for age, sex, ethnicity, and other potential confounders, patients treated with surgery had the lowest all-cause and lung cancer-specific mortality rates and had the highest 1-year survival and 5-year survival rates compared with the other treatment modalities in both the total and stratified populations, while those who did not receive treatment had the worst prognosis. In the total population, OS and lung cancer-specific survival were worse in the surgery plus radiotherapy group than in the surgery-only group, but better than in the radiotherapy-only group. The radiotherapy-only group had better OS and lung cancer-specific survival than the no treatment group.

In earlier studies, because of the increased surgical risks and postoperative complications in older patients, especially those over 80 years of age, many clinicians avoided surgery on the basis of age (6). As a result, older patients have been less likely than younger patients to undergo curative surgery for early-stage lung cancer (7–9). With the development of the economy, especially the digital economy, and the advances in medical care in recent years, the health status of the elderly in general is much better than in earlier generations and the risk of surgery has decreased (14). Many previous studies concluded that radiotherapy is more effective than surgery in this population. One study showed that radiotherapy is better tolerated for operable stage I NSCLC and can lead to better overall survival than surgery (10). This result might have been influenced by the surgical approach, because the majority of procedures in that study were more invasive open procedures. Minimally invasive thoracoscopic surgery reduces postoperative morbidity, shortens hospital stays, and is well tolerated by older patients (15). Moreover, the finding that radiotherapy is more effective than surgery might have to be reconsidered in today's world, where minimally invasive thoracoscopic surgery is widely available (16). In addition, the sample size included in that study was only 58 cases, which might not truly reflect the effect of the treatment modality. Our data came from the latest SEER database from 2010 to 2017, and included a sample of 7,045 cases. Our results showed that surgery had a more positive impact than radiotherapy in stage I and II NSCLC patients aged 80 years and older.

The efficacy of surgery in early-stage NSCLC was positive in the entire population; however, surgery is usually less suitable for older patients over 80 years of age because of their suspected frailty, higher risk of complications, or shorter “active” life expectancy (17). This distinction between younger patients and older patients over 80 years of age is not justified. Chest surgery should not be prohibited on the basis of age *per se*. For certain subgroups of older individuals, thoracic surgery has been proven to be a safe and practical choice (6, 17). For individuals with early-stage lung cancer, surgical interventions offer the best chance of recovery (18).

Our study found that those who opted for surgical treatment were younger, and younger patients tended to have fewer and less severe comorbidities and lower performance status (PS) scores, as well as more dominant frailty scores, resulting in better survival outcomes for this population, which might have introduced some selection bias into our findings. Geriatric oncology is often defined as “when the health status of the patient population begins to interfere with oncology decision guidelines (19).” This means that the biological age of an individual patient should be determined separately according to his or her individual PS and comorbidities, which will influence the decision, rather than a fixed age limit. High performance status scores and poor frailty scores are poor prognostic factors for lung cancer (20). In turn, weight loss, muscle wasting, immunosuppression, decreased endurance, and decreased free movement were associated with increased comorbidities. The most common comorbidities in patients with lung cancer are cardiovascular disease, chronic obstructive pulmonary disease, and anemia. Some studies have shown that the PS score and comorbidities are independent prognostic factors for lung cancer (21–26). Data on PS scores, frailty scores, and comorbidities might have yielded more accurate results if added to the study; however, the lack of these data in the SEER database prevented us from including these variables in further analysis. However, the absence of these data does not necessarily sway our conclusions. When patients can tolerate surgery, lobectomy remains the ideal surgical option for NSCLC; however, patients with co-morbidities are not without surgical options, and wedge resection offers adequate options for this population. This is particularly true for elderly patients, whose natural life expectancy is shorter than that of younger patients, and long-term survival becomes less important in this population compared to intermediate survival, under which circumstances wedge resection appears to offer similar rates of disease control. Therefore, patients with comorbidities are not necessarily less likely to undergo surgery than patients without co-morbidities, which might neutralize some of the bias associated with “younger patients are more likely to opt for surgery”. We hope that more detailed data will be available in the future to further corroborate our view.

Our study also found that all-cause mortality and lung cancer-specific mortality were higher in the surgery plus radiotherapy group than in the surgery-only group. Radiotherapy instead reduces the efficacy of surgery, which might be ascribed to the toxicity of radiotherapy. Radiotherapy-related toxicity can induce complications such as esophagitis, pneumonia, and pulmonary fibrosis (27). Our data were from the SEER database for the years 2010–2017.The SEER database lacks data on specific radiotherapy modalities; however, the population treated with radiotherapy in these years was more likely to have received conventional radiotherapy than the stereotactic body radiotherapy (SBRT), which has emerged in recent years. SBRT has been shown to provide short/medium term local control of the primary tumor comparable to surgery; therefore, SBRT has attracted a great deal of interest. Randomized data comparing surgery with SBRT are not available; however, a population-based paired comparison of SBRT with surgery in 120 elderly patients showed no difference in OS (1- and 3-year survival rates of 75% and 60% after surgery and 87% and 42% after SBRT, respectively) (28). In addition, an analysis of 10,923 patients who received five different treatments (lobectomy, sublobar resection, conventional radiotherapy, SBRT, and no treatment) showed similar OS after lobectomy and SBRT (17). SBRT is a breakthrough and landmark treatment achieved in radiotherapy in recent years, and perhaps the spread of SBRT could improve the survival of older patients with early-stage NSCLC. The availability of SBRT might improve the survival of elderly patients with early-stage NSCLC to a level close to that of surgical treatment, which needs to be further analyzed by obtaining more up-to-date data. However, in general, surgical treatment is more advantageous than radiotherapy for stage I and II elderly NSCLC patients.

Our competing risk model found that the surgery only group had the lowest lung cancer-related mortality, while there was no significant difference in non-lung cancer mortality between the treatment modalities, indicating that different treatment modalities did not affect the patients' risk of non-lung cancer death; however, surgical treatment significantly reduced patients' risk of lung cancer-specific death (29).

Another important factor influencing the outcome of the study was the surgical extent of resection. The absence of data on the extent of surgical resection in the SEER database prevented us from performing a stratified analysis based on the extent of surgical resection. For the past 50 years, lobectomy has been the standard of care for patients with early-stage lung cancer who can tolerate this procedure. However, lung-preserving procedures, such as segmental resection and wedge resection (Figure 2), are becoming more common in the management of lung cancer. In one study, only 16% of 243 patients aged less than 65 years were treated with wedge resection alone, compared with 63% of 40 patients aged 80 years or older(6). In addition, 28 of the 56 elderly patients (75 years or older) whose lung function was sufficient to tolerate lobectomy (FEV1 > 1 L) still received wedge resection as a definitive treatment. The operative mortality rate for wedge resection in elderly patients was zero, and only 1 of 52 patients (2.7%) underwent lobectomy. The mean postoperative hospital stay for elderly patients was 6 days after wedge resection and 8 days after lobectomy. After a median follow-up of 15 months, there was no difference in survival between the elderly patients who underwent wedge resection (median 38 months) and those who underwent lobectomy (median 35 months). The small or no difference in survival demonstrated by lobectomy vs. restrictive resection will not affect our conclusions. We hope to further confirm our conclusions with later studies comparing survival with different surgical resection scopes to the radiotherapy alone population.

One study showed that patients undergoing televised (TV) thoracoscopic-assisted lobectomy had approximately 2% complications and no reported perioperative deaths, compared with 7% complications and a 3.6% mortality rate in the open-chest surgery group (30). With the popularity of TV thoracoscopy-assisted surgery, the therapeutic advantages of surgery for patients with advanced early-stage NSCLC are more obvious compared with other treatment options. The lack of data on surgical modality in the SEER database prevented us from performing a stratified analysis based on surgical modality. The rapid growth in video-assisted thoracic surgery (VATS) occurred between 2010 and 2017, thus the surgical modality for the sample population was more likely to be VATS. VATS has been shown to lead to better survival than open surgery and might prove to be the preferred surgical procedure for older patients, who as a group, have more co-morbidities and are therefore at higher risk of developing more invasive surgical complications. These new surgical techniques might even reduce the incidence of postoperative complications in elderly patients, exposing them to less surgical and anesthetic stress, minimizing postoperative pain levels, and facilitating their faster return to the home environment. We concluded from the analysis of the results that the OS resulting from VATS treatment is better than that of radiotherapy alone in the early stage elderly NSCLC population; however, we do not know the survival comparison between open surgery and radiotherapy, and we hope that more data will be available in the future to allow a quantitative comparison of survival related to different surgical approaches compared with that of radiotherapy.

Another important variable is the resection margin status (R0, R1, or R2), which is not available in the SEER database; however, we can make some assumptions about the data. The National Comprehensive Cancer Network (NCCN) guidelines for surgical resection of NSCLC recommend negative margins, so most of our included patients who underwent surgery were more likely to have an R0 resection. Whether R1 resection and R2 resection would influence our conclusions will require verification using more data.

The absence of data on work status and comorbidities in the SEER database limited our ability to further compare patients in the surgical and radiotherapy groups. The choice of surgery is jointly influenced by physician recommendation and subjective decision making by the affected patient, and there is no formal treatment algorithm for patient selection; therefore, this might be a source of bias. In addition, our analysis was not stratified by type of surgical resection (wedge resection, segmental resection, lobectomy, or total lung resection) because of a lack of data on surgical modality. In addition, patients who received radiotherapy in this study were more likely to have received conventional radiotherapy rather than stereotactic radiotherapy, as there were no records of stereotactic radiotherapy approaches. Furthermore, the results of OS and lung cancer-specific survival in the surgery plus radiotherapy group in the stratified analysis were not stable across strata, which might have been caused by the insufficient sample size of the surgery plus radiotherapy group. In addition, the efficacy of the surgery plus radiotherapy group needs to be studied in a larger sample size. Finally, because of the inherent limitations of retrospective studies, the findings are inevitably biased and further prospective studies are needed to confirm these results.

## Conclusions

Surgery had a higher overall and lung cancer-specific survival rate than radiotherapy in the elderly early-stage NSCLC population. For advanced age patients with stage I and stage II NSCLC, surgical treatment might have a greater potential survival benefit.

## Data Availability

Publicly available datasets were analyzed in this study. This data can be found here: https://seer.cancer.gov/.
