# Supplementary material for: Surgery offers survival advantage over radiotherapy in patients who are 80 years and older with Stage I and II NSCLC: A retrospective cohort study of 7,045 patients
Source: Front Surg. 2022 Oct 4;9:1018320. doi: 10.3389/fsurg.2022.1018320 (PMC9577077; doi:10.3389/fsurg.2022.1018320)
Supplement: Supplementary file 1 [file Table1.docx]

**Table S1. Stratified analysis of the association between treatment method and mortality**

| **Sub-group**  **X= Treatment** | **N** | **Hazard Ratio (95% CI) *P*-value** | |
| --- | --- | --- | --- |
|  |  | **All-cause Mortality** | **Lung Cancer–Specific Mortality** |
| Age |  |  |  |
| Age = 80-84 years |  |  |  |
| Surgery only | 1689 | 1 | 1 |
| Radiation only | 1838 | 2.04 (1.84, 2.26) <0.0001 | 1.20 (1.09, 1.32) 0.0002 |
| Surgery +Radiation | 63 | 1.27 (0.92, 1.75) 0.1467 | 1.01 (0.72, 1.42) 0.9657 |
| No treatment | 770 | 4.19 (3.72, 4.71) <0.0001 | 1.72 (1.51, 1.97) <0.0001 |
| Age = 85-89 years |  |  |  |
| Surgery only | 424 | 1 | 1 |
| Radiation only | 1111 | 1.75 (1.49, 2.05) <0.0001 | 1.25 (1.07, 1.46) 0.0048 |
| Surgery +Radiation | 17 | 1.52 (0.86, 2.68) 0.1509 | 0.76 (0.37, 1.54) 0.444 |
| No treatment | 599 | 4.32 (3.64, 5.13) <0.0001 | 1.78 (1.47, 2.16) <0.0001 |
| Age = 90-94 years |  |  |  |
| Surgery only | 34 | 1 | 1 |
| Radiation only | 238 | 2.50 (1.47, 4.23) 0.0007 | 1.16 (0.73, 1.84) 0.5389 |
| Surgery +Radiation | 1 | 0.32 (0.04, 2.65) 0.2905 | 0.57 (0.06, 5.12) 0.6188 |
| No treatment | 191 | 6.35 (3.66, 11.02) <0.0001 | 2.41 (1.44, 4.04) 0.0008 |
| Race |  |  |  |
| Race = White |  |  |  |
| Surgery only | 1838 | 1 | 1 |
| Radiation only | 2759 | 1.95 (1.79, 2.13) <0.0001 | 1.23 (1.13, 1.34) <0.0001 |
| Surgery +Radiation | 71 | 1.33 (0.99, 1.79) 0.0572 | 0.88 (0.63, 1.22) 0.4448 |
| No treatment | 1313 | 4.23 (3.82, 4.67) <0.0001 | 1.76 (1.57, 1.97) <0.0001 |
| Race = Black |  |  |  |
| Surgery only | 113 | 1 | 1 |
| Radiation only | 197 | 2.53 (1.72, 3.71) <0.0001 | 1.62 (1.12, 2.34) 0.0112 |
| Surgery +Radiation | 2 | 0.95 (0.21, 4.33) 0.9472 | 1.10 (0.24, 5.07) 0.9017 |
| No treatment | 122 | 7.16 (4.73, 10.84) <0.0001 | 3.26 (2.05, 5.20) <0.0001 |
| Race = Other |  |  |  |
| Surgery only | 198 | 1 | 1 |
| Radiation only | 251 | 2.00 (1.43, 2.80) <0.0001 | 0.99 (0.74, 1.33) 0.9653 |
| Surgery +Radiation | 8 | 1.24 (0.47, 3.24) 0.6661 | 1.36 (0.50, 3.66) 0.5466 |
| No treatment | 173 | 4.85 (3.46, 6.79) <0.0001 | 1.43 (1.00, 2.04) 0.0494 |
| Sex |  |  |  |
| Sex = Male |  |  |  |
| Surgery only | 1042 | 1 | 1 |
| Radiation only | 1543 | 2.03 (1.80, 2.28) <0.0001 | 1.31 (1.17, 1.48) <0.0001 |
| Surgery +Radiation | 40 | 1.21 (0.82, 1.77) 0.335 | 0.78 (0.50, 1.23) 0.2857 |
| No treatment | 704 | 4.60 (4.02, 5.25) <0.0001 | 2.18 (1.87, 2.56) <0.0001 |
| Sex = Female |  |  |  |
| Surgery only | 1107 | 1 | 1 |
| Radiation only | 1664 | 1.89 (1.68, 2.13) <0.0001 | 1.14 (1.03, 1.27) 0.0156 |
| Surgery +Radiation | 41 | 1.42 (0.95, 2.11) 0.0868 | 1.08 (0.72, 1.63) 0.7181 |
| No treatment | 904 | 4.19 (3.68, 4.77) <0.0001 | 1.52 (1.32, 1.75) <0.0001 |
| Year of diagnosis |  |  |  |
| Year of diagnosis = 2010-2013 |  |  |  |
| Surgery only | 1200 | 1 | 1 |
| Radiation only | 1365 | 1.97 (1.78, 2.18) <0.0001 | 1.36 (1.20, 1.54) <0.0001 |
| Surgery +Radiation | 47 | 1.39 (1.01, 1.92) 0.0434 | 0.88 (0.55, 1.39) 0.5757 |
| No treatment | 856 | 3.93 (3.50, 4.42) <0.0001 | 2.06 (1.77, 2.41) <0.0001 |
| Year of diagnosis = 2014-2017 |  |  |  |
| Surgery only | 949 | 1 | 1 |
| Radiation only | 1842 | 2.07 (1.79, 2.39) <0.0001 | 1.13 (1.02, 1.25) 0.0246 |
| Surgery +Radiation | 34 | 1.07 (0.62, 1.83) 0.8119 | 0.89 (0.60, 1.34) 0.5851 |
| No treatment | 752 | 5.36 (4.58, 6.29) <0.0001 | 1.52 (1.31, 1.75) <0.0001 |
| Primary site |  |  |  |
| Primary site = Upper lobe |  |  |  |
| Surgery only | 1202 | 1 | 1 |
| Radiation only | 1849 | 1.88 (1.68, 2.10) <0.0001 | 1.23 (1.10, 1.36) 0.0001 |
| Surgery +Radiation | 39 | 1.00 (0.66, 1.53) 0.9883 | 0.81 (0.53, 1.24) 0.3308 |
| No treatment | 908 | 4.56 (4.03, 5.17) <0.0001 | 1.86 (1.62, 2.14) <0.0001 |
| Primary site = Lower lobe |  |  |  |
| Surgery only | 771 | 1 | 1 |
| Radiation only | 1124 | 2.15 (1.87, 2.47) <0.0001 | 1.22 (1.07, 1.40) 0.0035 |
| Surgery +Radiation | 36 | 1.41 (0.94, 2.13) 0.0996 | 1.13 (0.73, 1.76) 0.5856 |
| No treatment | 527 | 4.33 (3.71, 5.06) <0.0001 | 1.79 (1.50, 2.13) <0.0001 |
| Primary site = Main bronchus |  |  |  |
| Surgery only | 3 | 1 | 1 |
| Radiation only | 26 | 0.01 (0.00, 0.16) 0.0016 | 0.00 (0.00, 0.00) <0.0001 |
| Surgery +Radiation | 1 | 0.04 (0.00, 2.55) 0.1284 | 0.04 (0.00, Inf) 0.9998 |
| No treatment | 23 | 0.07 (0.01, 0.85) 0.037 | 0.00 (0.00, 0.00) <0.0001 |
| Primary site = Unknow |  |  |  |
| Surgery only | 24 | 1 | 1 |
| Radiation only | 65 | 1.17 (0.55, 2.50) 0.6819 | 2.41 (0.81, 7.17) 0.1123 |
| No treatment | 73 | 3.85 (1.80, 8.25) 0.0005 | 2.81 (0.81, 9.72) 0.1026 |
| Grade |  |  |  |
| Grade = I |  |  |  |
| Surgery only | 430 | 1 | 1 |
| Radiation only | 291 | 1.90 (1.50, 2.41) <0.0001 | 1.16 (0.96, 1.39) 0.1235 |
| Surgery +Radiation | 8 | 2.23 (0.90, 5.54) 0.0843 | 0.68 (0.21, 2.16) 0.5135 |
| No treatment | 146 | 4.14 (3.16, 5.41) <0.0001 | 1.43 (1.09, 1.89) 0.01 |
| Grade = II |  |  |  |
| Surgery only | 960 | 1 | 1 |
| Radiation only | 649 | 1.94 (1.68, 2.23) <0.0001 | 1.23 (1.07, 1.42) 0.0037 |
| Surgery +Radiation | 32 | 1.28 (0.83, 1.97) 0.2713 | 1.02 (0.64, 1.64) 0.9375 |
| No treatment | 206 | 4.20 (3.50, 5.04) <0.0001 | 1.54 (1.20, 1.97) 0.0007 |
| Grade = III |  |  |  |
| Surgery only | 538 | 1 | 1 |
| Radiation only | 630 | 2.11 (1.81, 2.47) <0.0001 | 1.37 (1.16, 1.62) 0.0003 |
| Surgery +Radiation | 33 | 1.28 (0.82, 1.99) 0.2822 | 1.06 (0.64, 1.76) 0.8145 |
| No treatment | 238 | 5.39 (4.45, 6.53) <0.0001 | 2.27 (1.69, 3.05) <0.0001 |
| Grade = IV |  |  |  |
| Surgery only | 20 | 1 | 1 |
| Radiation only | 14 | 5.31 (2.47, 11.39) <0.0001 | 8.93 (0.45, 176.28) 0.1502 |
| No treatment | 11 | 12.30 (5.32, 28.43) <0.0001 | 19.01 (0.84, 430.52) 0.0643 |
| Grade = Unknow |  |  |  |
| Surgery only | 201 | 1 | 1 |
| Radiation only | 1623 | 1.52 (1.24, 1.88) <0.0001 | 1.13 (0.95, 1.35) 0.1603 |
| Surgery +Radiation | 8 | 1.04 (0.42, 2.57) 0.9278 | 0.78 (0.34, 1.78) 0.5547 |
| No treatment | 1007 | 3.29 (2.66, 4.07) <0.0001 | 1.71 (1.42, 2.07) <0.0001 |
| Laterality |  |  |  |
| Laterality = Left- origin of primary |  |  |  |
| Surgery only | 894 | 1 | 1 |
| Radiation only | 1436 | 1.90 (1.68, 2.15) <0.0001 | 1.28 (1.14, 1.45) <0.0001 |
| Surgery +Radiation | 33 | 1.06 (0.66, 1.71) 0.7998 | 1.08 (0.70, 1.68) 0.7247 |
| No treatment | 712 | 4.72 (4.09, 5.43) <0.0001 | 2.11 (1.80, 2.48) <0.0001 |
| Laterality = Right- origin of primary |  |  |  |
| Surgery only | 1255 | 1 | 1 |
| Radiation only | 1769 | 2.01 (1.80, 2.25) <0.0001 | 1.17 (1.05, 1.30) 0.0034 |
| Surgery +Radiation | 48 | 1.39 (0.99, 1.95) 0.0595 | 0.82 (0.54, 1.25) 0.3554 |
| No treatment | 892 | 4.16 (3.67, 4.71) <0.0001 | 1.59 (1.39, 1.83) <0.0001 |
| Histology |  |  |  |
| Histology = Squamous cell neoplasms |  |  |  |
| Surgery only | 537 | 1 | 1 |
| Radiation only | 1088 | 1.85 (1.61, 2.14) <0.0001 | 1.29 (1.11, 1.51) 0.0009 |
| Surgery +Radiation | 24 | 0.96 (0.58, 1.60) 0.883 | 0.81 (0.45, 1.47) 0.4942 |
| No treatment | 418 | 5.06 (4.30, 5.96) <0.0001 | 2.18 (1.74, 2.72) <0.0001 |
| Histology = Adenomas and adenocarcinomas |  |  |  |
| Surgery only | 1221 | 1 | 1 |
| Radiation only | 1529 | 2.09 (1.86, 2.35) <0.0001 | 1.21 (1.08, 1.34) 0.0006 |
| Surgery +Radiation | 48 | 1.77 (1.25, 2.51) 0.0013 | 1.04 (0.70, 1.54) 0.8507 |
| No treatment | 693 | 3.49 (3.05, 4.00) <0.0001 | 1.38 (1.20, 1.59) <0.0001 |
| Histology = Other |  |  |  |
| Surgery only | 391 | 1 | 1 |
| Radiation only | 590 | 1.93 (1.54, 2.42) <0.0001 | 1.29 (1.05, 1.59) 0.0162 |
| Surgery +Radiation | 9 | 0.75 (0.28, 2.02) 0.5649 | 0.67 (0.30, 1.53) 0.3435 |
| No treatment | 497 | 5.31 (4.15, 6.79) <0.0001 | 2.79 (2.16, 3.59) <0.0001 |
| Stage |  |  |  |
| Stage = I |  |  |  |
| Surgery only | 1690 | 1 | 1 |
| Radiation only | 2514 | 1.78 (1.62, 1.96) <0.0001 | 1.23 (1.13, 1.35) <0.0001 |
| Surgery +Radiation | 35 | 1.19 (0.76, 1.85) 0.4477 | 0.76 (0.50, 1.16) 0.1998 |
| No treatment | 1032 | 4.07 (3.63, 4.55) <0.0001 | 1.74 (1.55, 1.95) <0.0001 |
| Stage = II |  |  |  |
| Surgery only | 459 | 1 | 1 |
| Radiation only | 693 | 2.20 (1.86, 2.59) <0.0001 | 1.14 (0.93, 1.39) 0.2155 |
| Surgery +Radiation | 46 | 1.62 (1.13, 2.33) 0.0089 | 1.23 (0.79, 1.92) 0.3655 |
| No treatment | 576 | 4.46 (3.76, 5.29) <0.0001 | 1.85 (1.47, 2.32) <0.0001 |
| Sequence number |  |  |  |
| Sequence number = First/only primary |  |  |  |
| Surgery only | 1216 | 1 | 1 |
| Radiation only | 1768 | 1.93 (1.72, 2.16) <0.0001 | 1.24 (1.11, 1.37) <0.0001 |
| Surgery +Radiation | 49 | 1.44 (1.01, 2.07) 0.0447 | 0.93 (0.62, 1.40) 0.7345 |
| No treatment | 968 | 4.77 (4.21, 5.40) <0.0001 | 1.94 (1.69, 2.24) <0.0001 |
| Sequence number = Second/higher- order primary |  |  |  |
| Surgery only | 933 | 1 | 1 |
| Radiation only | 1439 | 2.04 (1.80, 2.31) <0.0001 | 1.22 (1.08, 1.38) 0.0011 |
| Surgery +Radiation | 32 | 1.14 (0.74, 1.75) 0.5583 | 0.94 (0.60, 1.48) 0.8044 |
| No treatment | 640 | 3.89 (3.38, 4.48) <0.0001 | 1.60 (1.37, 1.88) <0.0001 |
| Number of tumors |  |  |  |
| Number of tumors = 1 |  |  |  |
| Surgery only | 1021 | 1 | 1 |
| Radiation only | 1568 | 1.96 (1.73, 2.21) <0.0001 | 1.22 (1.09, 1.37) 0.0008 |
| Surgery +Radiation | 42 | 1.63 (1.11, 2.39) 0.0134 | 1.07 (0.69, 1.68) 0.7535 |
| No treatment | 895 | 4.84 (4.24, 5.53) <0.0001 | 1.89 (1.63, 2.19) <0.0001 |
| Number of tumors = 2 |  |  |  |
| Surgery only | 739 | 1 | 1 |
| Radiation only | 1055 | 2.05 (1.78, 2.37) <0.0001 | 1.23 (1.07, 1.41) 0.0041 |
| Surgery +Radiation | 27 | 0.98 (0.60, 1.57) 0.9186 | 0.82 (0.49, 1.38) 0.4535 |
| No treatment | 488 | 3.92 (3.33, 4.62) <0.0001 | 1.58 (1.31, 1.90) <0.0001 |
| Number of tumors = 3+ |  |  |  |
| Surgery only | 389 | 1 | 1 |
| Radiation only | 584 | 1.94 (1.60, 2.35) <0.0001 | 1.28 (1.06, 1.54) 0.0087 |
| Surgery +Radiation | 12 | 1.30 (0.64, 2.65) 0.4714 | 0.95 (0.49, 1.87) 0.8858 |
| No treatment | 225 | 3.93 (3.12, 4.94) <0.0001 | 1.93 (1.51, 2.46) <0.0001 |
| Marital status |  |  |  |
| Marital status = Married |  |  |  |
| Surgery only | 1072 | 1 | 1 |
| Radiation only | 1331 | 2.07 (1.83, 2.34) <0.0001 | 1.25 (1.11, 1.40) 0.0003 |
| Surgery +Radiation | 45 | 1.11 (0.74, 1.67) 0.6064 | 0.90 (0.60, 1.33) 0.5917 |
| No treatment | 614 | 4.49 (3.89, 5.17) <0.0001 | 1.83 (1.56, 2.14) <0.0001 |
| Marital status = Widowed |  |  |  |
| Surgery only | 733 | 1 | 1 |
| Radiation only | 1287 | 1.99 (1.73, 2.27) <0.0001 | 1.25 (1.10, 1.42) 0.0007 |
| Surgery +Radiation | 27 | 1.55 (0.99, 2.42) 0.0557 | 1.03 (0.60, 1.77) 0.9021 |
| No treatment | 688 | 4.50 (3.88, 5.23) <0.0001 | 1.73 (1.46, 2.04) <0.0001 |
| Marital status = Other |  |  |  |
| Surgery only | 344 | 1 | 1 |
| Radiation only | 589 | 1.71 (1.38, 2.10) <0.0001 | 1.13 (0.93, 1.38) 0.2149 |
| Surgery +Radiation | 9 | 1.35 (0.65, 2.83) 0.423 | 0.59 (0.21, 1.62) 0.3063 |
| No treatment | 306 | 4.02 (3.19, 5.08) <0.0001 | 1.82 (1.41, 2.35) <0.0001 |
| Chemotherapy |  |  |  |
| Chemotherapy = No |  |  |  |
| Surgery only | 2061 | 1 | 1 |
| Radiation only | 2879 | 1.97 (1.81, 2.15) <0.0001 | 1.25 (1.15, 1.36) <0.0001 |
| Surgery +Radiation | 57 | 1.06 (0.75, 1.48) 0.7451 | 0.82 (0.58, 1.15) 0.2482 |
| No treatment | 1489 | 4.45 (4.04, 4.90) <0.0001 | 1.81 (1.63, 2.02) <0.0001 |
| Chemotherapy = Yes |  |  |  |
| Surgery only | 88 | 1 | 1 |
| Radiation only | 328 | 2.02 (1.40, 2.89) 0.0001 | 0.87 (0.58, 1.29) 0.474 |
| Surgery +Radiation | 24 | 2.19 (1.25, 3.85) 0.0062 | 1.40 (0.70, 2.78) 0.3377 |
| No treatment | 119 | 2.90 (1.94, 4.33) <0.0001 | 1.20 (0.75, 1.90) 0.4516 |

Note: Stratification adjusted for all factors (age, race, sex, year of diagnosis, primary site, grade, laterality, histology, stage group, sequence number, number of tumors, marital status, chemotherapy), except the Stratification factor itself.
